# Calcium-Dependent Translocation of S100B Is Facilitated by Neurocalcin Delta

**DOI:** 10.3390/molecules26010227

**Published:** 2021-01-05

**Authors:** Jingyi Zhang, Anuradha Krishnan, Hao Wu, Venkat Venkataraman

**Affiliations:** 1 Department of Cell Biology and Neuroscience, Graduate School of Biomedical Sciences, School of Osteopathic Medicine, Rowan University, Stratford, NJ 08084, USA; serenazjy@gmail.com (J.Z.); krishnan@rowan.edu (A.K.); haowubiomed@gmail.com (H.W.); 2 Department of Rehabilitation Medicine, NeuroMusculoskeletal Institute, School of Osteopathic Medicine, Rowan University, Stratford, NJ 08084, USA

**Keywords:** S100B, neurocalcin delta, hippocalcin, calcium, translocation

## Abstract

S100B is a calcium-binding protein that governs calcium-mediated responses in a variety of cells—especially neuronal and glial cells. It is also extensively investigated as a potential biomarker for several disease conditions, especially neurodegenerative ones. In order to establish S100B as a viable pharmaceutical target, it is critical to understand its mechanistic role in signaling pathways and its interacting partners. In this report, we provide evidence to support a calcium-regulated interaction between S100B and the neuronal calcium sensor protein, neurocalcin delta both in vitro and in living cells. Membrane overlay assays were used to test the interaction between purified proteins in vitro and bimolecular fluorescence complementation assays, for interactions in living cells. Added calcium is essential for interaction in vitro; however, in living cells, calcium elevation causes translocation of the NCALD-S100B complex to the membrane-rich, perinuclear trans-Golgi network in COS7 cells, suggesting that the response is independent of specialized structures/molecules found in neuronal/glial cells. Similar results are also observed with hippocalcin, a closely related paralog; however, the interaction appears less robust in vitro. The N-terminal region of NCALD and HPCA appear to be critical for interaction with S100B based on in vitro experiments. The possible physiological significance of this interaction is discussed.

## 1. Introduction

Calcium is a versatile second messenger that plays a pivotal role in a variety of physiological processes. Changes in intracellular calcium concentration can trigger multiple, diverse signaling pathways and processes ranging from fertilization to muscle contraction under normal conditions and neurodegenerative diseases and cancer in pathological states. The tight spatial and temporal regulation of responses to changes in intracellular calcium, arguably, is of the greatest significance in modulating neuronal activity. Such modulation occurs directly within neurons and indirectly through astrocyte/glial pathways.

Ever since the identification of neuroglia by Rudolph Virchow [1] and astrocyte independently by Michael von Lenhosseck, Kolliker and Anderiezen (reviewed in: [2]), as part of the brain, their role has been shifting towards more active participation in neuronal activity rather than passive support. While the effect of calcium signaling within the neurons on modulating neuronal activity has been well-established and accepted, the role for the astrocyte/glial signaling has gone through waves of acceptance and questioning [3]. It is suggested that two aspects may account for observed discrepancies: the hormetic response (opposing effects based on concentration; reviewed in [4,5,6]) elicited by calcium and subcellular compartmentalization.

S100B, primarily astrocytic in origin, is a unique signaling molecule that impacts multiple signaling pathways in a hormetic fashion—sometimes negatively, sometimes positively [7,8]. The S100 family now includes more than 20 genes paralogous to S100B, with functions in healthy and diseased states [9]; however, the focus for this report is S100B. S100B is a homodimer of a 92-amino acid protein, termed S100b, with a molecular mass of 10,713 Da, but migrates between 9 and 14 kDa on SDS-polyacrylamide gels. Crystal structures and refined NMR structures [10,11] reveal that the monomer contains two EF-hands—one non-canonical and one conventional—that bind calcium. S100B can bind calcium and undergo a conformational change, which allows it to control cellular activity by interacting with other proteins—a feature of most S100 proteins (reviewed in [12]). However, these conformational changes are unlike other more conventional EF-containing proteins and are unique for S100B protein [13]. The neuronal calcium sensor (NCS) proteins, for example, have a relatively hydrophobic N-terminal region (often myristoylated) that is buried in the calcium-free state and is exposed upon binding of calcium. This exposed region then interacts with target proteins—sometimes additional regions are also involved. In contrast to this largely hydrophobic interacting surface in NCS proteins, S100B surface is abundantly populated with charged residues—both negative and positive; in addition, the hydrophobic interfacial cleft is also exposed upon calcium binding. Thus, a broader and flatter surface for interaction—ionic and hydrophobic—explains the ability to bind to a variety of proteins, further confounding the ability to predict interacting surfaces based on protein sequence alone [12,14,15]. The difference between the conformational changes in NCS proteins and S100B has also been demonstrated experimentally through calcium-induced mobility shift assays [13]: binding to calcium results in a mobility shift in the NCS proteins, but no such shift is observed with S100B. Even among the S100 family, S100B appears to be uniquely designed for interactions. Thus, the S100B protein is uniquely specialized to sense changes in calcium levels and mediates appropriate responses, especially in the nervous system.

Expression of S100B in the nervous system is primarily in the glial cells—astrocytes in the central nervous system and Schwann cells in the peripheral nervous system, where they carry out intracellular and extracellular functions (reviewed in [7,8,16]). S100B functions as a modulator of protein phosphorylation, enzyme activation and calcium homeostasis intracellularly; it also alters transcription impacting cell proliferation and migration, apoptosis and differentiation extracellularly. These functions are mediated primarily through RAGE (Receptor for Advanced Glycation Endproducts) and Toll-Like Receptors (TLRs)—known to date. At the organism level, S100B contributes to a range of processes from neurodegeneration to cardiomyocyte remodeling, from melanomas to gliomas. Since these functions are relevant to both healthy and diseased states and, since S100B is detectable in human serum and cerebrospinal fluid (CSF), a focused effort has been under way to determine if S100B could serve as a biomarker for various diseases (reviewed in: [17,18,19,20,21,22,23]) and a therapeutic target (reviewed in: [17,24,25,26,27,28]). However, understanding the mechanism of action is critical in determining the validity of a pharmaceutical target for therapy.

The dual nature of action—intra- and extracellular—by S100B (reviewed in: [7,16,29]) poses a significant challenge in delineating its precise mechanism of action in many instances. Further challenges are imposed by the compartmentalization of S100B expression and its interacting partners. For example, in the retina, it has been demonstrated that S100B is expressed and stimulates the rod outer segment membrane guanylate cyclase (ROS-GC); however, differences in the biochemical properties of the protein [30,31,32] and the histochemical localization within the retina have been observed [31,33,34]. In order to develop effective strategies of using S100B as a therapeutic/pharmacological target, it is essential to examine its subcellular localization and interacting partners—especially in the context of changing intracellular calcium concentrations. In this report, interaction of S100B with another neuronal calcium sensor protein, neurocalcin delta (NCALD) has been characterized both in vitro as well asin living cells in culture, and the response to changing intracellular calcium levels has been documented. In addition, possible binding of S100B to hippocalcin (HPCA)—a close homolog of NCALD—has also been similarly analyzed.

## 2. Results

### 2.1. NCALD and S100B Interact In Vitro

**Figure 1:**
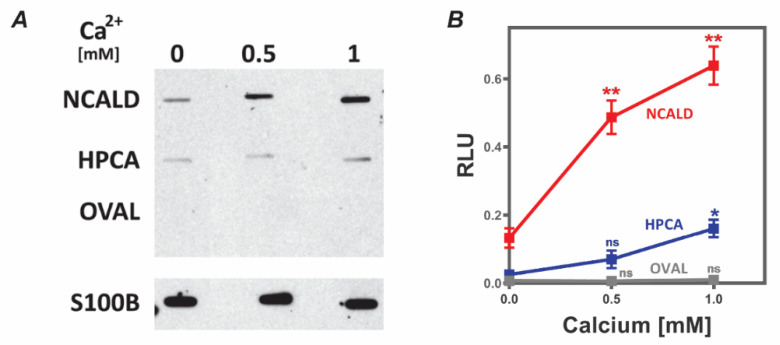
NCALD binds to S100B in vitro: Purified neurocalcin delta (NCALD), hippocalcin (HPCA) and commercial ovalbumin (OVAL) were blotted onto nitrocellulose and the membrane was then incubated with S100B in the presence of indicated concentrations of CaCl_2_. After 1 h incubation, the blot was washed with buffer. Bound S100B was detected using its antibody following standard Western blotting protocol and ECL detection. (**A**) A representative blot. (**B**) Data provided was collected from at least five independent experiments with three different preparations of the proteins. Intensity for each band was measured over the background using imageJ and then plotted (mean ± SEM) as relative luminescence unit (RLU) in Prism (9.0). Student’s *t*-test was carried out for each protein to determine significance compared to values obtained at 0 mM calcium. ns *p* > 0.01; * *p* ≤ 0.05; ** *p* ≤ 0.01.

It has been suggested that S100B is one of the target proteins of NCALD and that the S100B-NCALD complex may be involved in Ca^2+^ signaling in the glial cell [35]. Therefore, it was of interest to determine if NCALD and S100B bind and if they did, whether the binding contributed to the cellular response to calcium signals. As a first step, purified S100B and NCALD were used for in vitro binding assays through membrane overlay. Hippocalcin (HPCA) is another neuronal calcium sensor protein within the VILIP family, which shares 88% identity (95% similarity) at the primary amino acid sequence level with NCALD [36]. Therefore, HPCA was also used to test binding as described in “Materials and Methods”. An additional protein that does not bind calcium, ovalbumin, was used as a control. The results are presented in Figure 1.

The results suggest that S100B binds to both NCALD and HPCA, but better to NCALD than HPCA. There is no binding to ovalbumin. Furthermore, the binding of S100B to NCALD and HPCA is facilitated by calcium.

### 2.2. Binding to S100B Requires the N-Terminal Region of NCALD that Houses EF1 Hand

**Figure 2:**
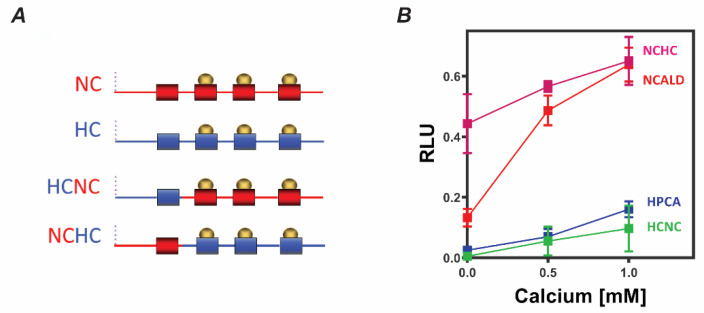
The N-terminal region of NCALD is necessary for the binding to S100B in vitro: (**A**) Chimeric constructs of NCALD and HPCA were created where the N-terminal region including the EF1 hand was swapped between the two proteins. EF hands are represented by filled rectangles. NCHC, for example, carried the N-terminal region of NCALD with the remaining portion of HPCA (**B**) Purified NCALD, HPCA, NCHC and HCNC were spotted onto nitrocellulose and the membrane was then incubated with S100B in the presence of indicated concentrations of CaCl_2_. Binding and analyses were carried out as indicated earlier. Data presented (mean ± SEM) was obtained from at least five independent experiments with three different protein preparations.

NCALD and HPCA share 88% amino acid identity and 95% similarity yet exhibited differential binding to S100B. To further investigate the binding site, we created chimeric proteins: NCHC, which contains the N-terminal region of NCALD encompassing the EF1 hand, but the remaining three EF hands of HPCA; HCNC, which contains the corresponding region from HPCA, but the remaining three EF hands of NCALD (Figure 2A). Purified NCHC and HCNC were tested in membrane overlay. The results showed (Figure 2B) that NCHC binding to S100B is similar to NCALD, while HCNC binding is comparable to that of HPCA. The observation suggests that the N-terminal region including the EF hand 1—the most divergent region between NCALD and HPCA—constitutes the binding site for S100B in both the proteins. It is noted that the calcium-dependence of binding is less robust in NCHC when compared to NCALD. A similar result, albeit less striking, is also observable with HCNC when compared to HPCA. It remains to be explored whether this is due to the altered binding or altered calcium-sensing in the chimeric proteins.

### 2.3. NCALD and S100B Interact in Living Cells

**Figure 3:**
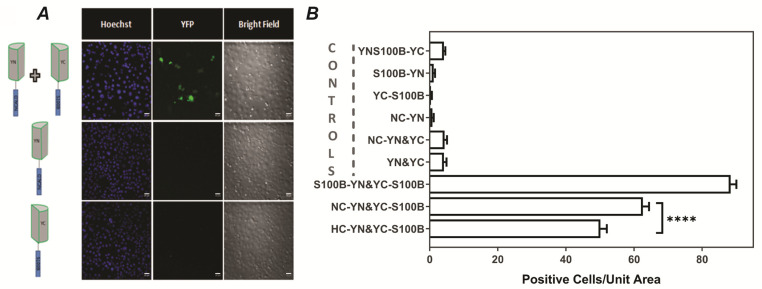
NCALD and S100B interact in living cells: COS 7 cells were cotransfected with constructs NCALD-YN-pcDNA1 and YC-S100B-pcDNA1. Cotransfection of S100B-YN and YC-S100B served as positive control while negative controls include transfection of S100B-YN, YC-S100B, NCALD-YN, YN&S100B-YC, NCALD-YN&YC and YN&YC. Cells were monitored by using Nikon Confocal microscope 48 hours after transfection. (**A**) Images of transfected cells with vectors individually or together (**B**) Analysis of complex formation: Positive cells numbers per unit area were obtained. Data from six independent transfections were pooled for controls; for S100B-S100B, NCALD-S100B and HPCA-S100B, data from twenty independent transfections were pooled and plotted in Prism (9.0). **** *p* ≤ 0.0001. Scale bar = 50 μm.

The results presented above show that NCALD and S100B bind specifically and the interaction requires the N-terminal region of NCALD. The next step was to investigate if the interaction occurred in living cells. To accomplish this purpose, we used bimolecular fluorescence complementation (BiFC) assay, based on the tagging of two proteins with half of a fluorescent protein (FP) each [37,38]. Upon the interaction of the putative binding partners, the two halves of the fluorescent protein are brought closer, associate with each other and form the functional protein that fluoresces (Figure 3A) [37,38]. In order to investigate the interaction between NCALD and S100B, we fused NCALD and S100B with YFP 1-158 (YN) and YFP 159-238(YC) respectively. Cotransfection with NCALD-YN-pcDNA1 and YC-S100B-pcDNA1 into COS7 cells generated fluorescent signal while transfection of individual constructs alone did not (Figure 3A). The active form of S100B is known to be a dimer. Therefore, we fused S100B with YFP 1-158(YN) and YFP 159-238(YC) and transfected the two constructs into COS7 cells as a positive control. Cotransfection of S100B-YN with YC-S100B into COS7 cells also generated the fluorescent signal. Multiple negative controls including S100B-YN, YC-S100B, NCALD-YN, YN&S100B-YC, NCALD-YN&YC and YN&YC were also tested. The results presented (Figure 3B) show that specific interaction was observed only between monomeric units of S100B, between S100B and NCALD and between S100B and HPCA. Furthermore, based on positive cells/unit area, the results show statistically significant differences between NCALD-S100B and HPCA-S100B. It remains to be determined if these differences are indicative of different affinities for the interacting pairs, as the in vitro data for NCALD and HPCA suggest (Figure 1 and Figure 2). Nevertheless, the deliberate choice of a non-neuronal, commonly used cell line and the observations underscore the possible ubiquitous nature of these interactions, even in the absence of specialized structures in astrocytes and neurons and specific proteins expressed therein. Another noteworthy difference is the calcium-dependence of the interaction between S100B and its interacting partner: the BiFC experiments demonstrate that interactions were observed after transfection without manipulating the intracellular calcium levels. Based on the rather high calcium concentrations needed to promote interactions in vitro, there are two possible explanations: (1) the interactions within cells are much more stable and robust compared to in vitro and (2) the endogenous calcium levels in COS7 cells are sufficient to promote the interactions. Therefore, it was of interest to investigate the effect, if any, of manipulating intracellular calcium concentrations on these interactions in this scenario.

### 2.4. Translocation of the Complexes in Response to Change in Calcium

Members of NCS proteins are myristoylated at the N-terminus by N-myristoyl transferase. The myristoyl group allows for the interaction with hydrophobic fragments, which facilitates translocation from the cytosol to membrane fractions [39]. Using live imaging in HeLa cells, translocation of HPCA (fused with fluorescent proteins) into the highly membranous, perinuclear, trans-Golgi network (TGN) region has been demonstrated in response to elevated intracellular calcium [40,41].

**Figure 4:**
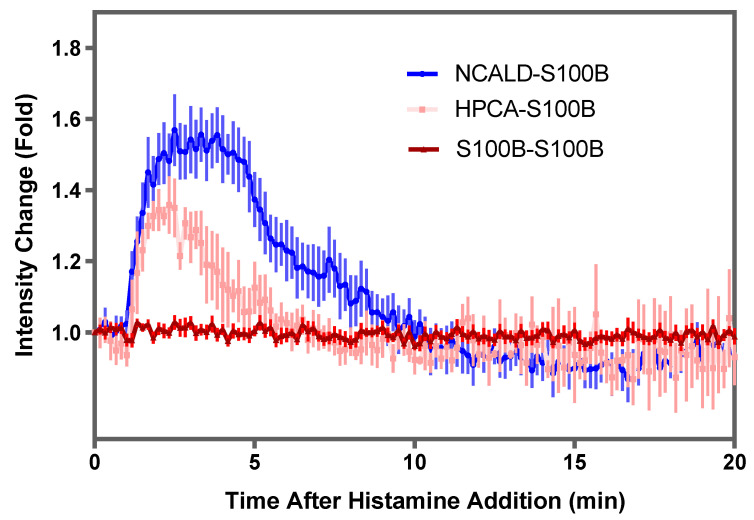
Translocation kinetics of S100B complexes—determination of a change in fluorescent intensities as a function of time: Readings from several experiments were averaged and the mean ± SEM is presented for the fold-change in fluorescent intensity observed in the trans-Golgi network (TGN) region for cells transfected with YN-NCALD and YC-S100B (NCALD-S100B, blue line, *n* = 20 cells); YN-HPCA and YC-S100B (HPCA-S100B, pink lines, *n* = 10 cells) and YN-S100B and YC-S100B (S100B-S100B, red line, *n* = 20 cells). At least three independent transfections were used to collect the data.

We adopted a similar approach to investigate the behavior of the complexes—NCALD-S100B, HPCA-S100B and S100B-S100B. We chose the COS7 cell system, which does not express S100B, NCALD or HPCA and is also not a specialized neuronal or astrocytic cell; on the other hand it boasts superior morphology of the endoplasmic reticulum and responds to histamine treatment with altered calcium [42,43]; treatment with histamine, a known physiological modulator, has also been documented to influence translocation of HPCA [41]. More analytical details are presented in Supplementary Materials and the results are summarized in Figure 4. Under normal conditions, fluorescence in transfected cells from all three complexes—NCALD-S100B, HPCA-S100B and S100B-S100B—were found to be diffused throughout the cell. After histamine addition, NCALD-S100B and HPCA-S100B translocated and localized within a specific perinuclear region of the cell rich in trans-Golgi network (TGN) membranes. In contrast, no significant translocation was observable upon histamine addition in cells transfected with S100B-S100B complex. The peak intensity for the translocated NCALD-S100B complex was reached at 3.15 ± 0.197 min (*n* = 20) and for the HPCA-S100B complex, at 2.25 ± 0.443 min (*n* = 10) after histamine addition. Based on assessment of calcium transients with cotransfected RCaMP, the maximum calcium concentration was reached at 2.11 ± 0.54 min (*n* = 8) after histamine addition; this value is comparable to about 1.66 min estimated earlier [43]. Over time, both HPCA-S100B and NCALD-S100B complexes diffused out of the region of interest (Figure 4). However, the kinetics was different: S100B complexed with NCALD stayed at the perinuclear region substantially longer than that complexed with HPCA (Figure 4). The observation suggests that the NCALD-S100B complex may have a broader range of sensitivity to calcium and/or a higher affinity to the TGN when compared to the HPCA-S100B complex.

## 3. Discussion

In this report, we demonstrated that S100B binds NCALD both in vitro and in living cells and identify that the N-terminal region of NCALD that includes the EF1 hand is essential for this binding. Furthermore, the NCALD-S100B complex translocates upon elevation in cellular calcium concentration while the S100B-S100B complex does not. The observations suggest that NCALD serves as a calcium-dependent chaperon for translocating S100B in living cells. It would explain how S100B, mostly found in the cytoplasm, could target proteins in membranous compartments, such as ROSGC1 [31]. While we have focused on the TGN area, it is likely that the complex also translocates to other membranous regions in the cell. As discussed earlier, properties of the S100B protein are not favorable to large hydrophobic interactions after calcium binding; however, those of NCALD are. Thus, a unique and elegant partnering system is created where a normally cytosolic protein, S100B, binds to another cytosolic protein, NCALD, which enables the complex to be translocated to membrane compartments. A similar role for HPCA might also be envisioned, although the in vitro experiments indicate that its interaction with S100B is weaker compared to NCALD—an observation substantiated by results obtained in cultured cells (Figure 3B). For this reason, we placed more emphasis on the S100B-NCALD interaction.

NCALD, like S100B, is a calcium-sensor protein and is a member of the neuronal calcium sensor (NCS) protein family. It is primarily expressed in the central nervous system, spinal cord, retina, inner ear, olfactory epithelium and zona glomerulosa of the adrenal gland, to name a few [44,45]. It consists of four EF hands although EF1 hand is disabled from binding calcium. A major feature of NCALD is N-terminal myristoylation, which allows NCALD to interact with cell membrane. The myristoyl group is sequestered in the Ca^2+^ free state in the protein. When it binds to Ca^2+^, the myristoyl group extrudes, allowing it to interact with the membrane—often described as the calcium-myristoyl switch [44,46]. A major consequence of calcium-myristoyl switch is the exposure of the buried N-terminus in the calcium-bound state; as a result, the myristoyl chain covalently attached to the N-terminus is also exposed targeting NCALD (and HPCA) to membranous cellular compartments—a feature found in other NCS proteins also [40,41]. Upon elevation of intracellular calcium, NCALD translocates to the perinuclear compartment from cytosol [40]. Another consequence of the calcium-myristoyl switch could also be the exposure of the binding sites located in the N-terminal region to target proteins. Our observations in vitro—that S100B binding to NCALD is calcium-dependent and the binding is mediated by the N-terminal region of NCALD—are consistent with this explanation. It is noted that the conformational changes due to the calcium-myristoyl switch may be assayed in vitro using the calcium induced mobility shift assay (CIMSA) [13]. Further analysis is in progress. 

There is direct evidence that the two proteins are expressed in a colocalized fashion in glial cells [35], and different types of neurons, for example, in the Purkinje cells of the cerebellum [47]. Both NCALD and S100B have been independently localized to identical regions in the retina [31,33,48]. What could be the physiological significance of this interaction? This report suggests that NCALD (and potentially HPCA) serves as a calcium-dependent chaperone for S100B. As in the retina, NCALD could enable delivery of S100B to common targets—such as ROSGC1, located on the cell membrane—in response to calcium. Interestingly, S100B and NCALD have non-overlapping binding sites on ROSGC1 and, together, may cause a greater stimulation of the catalytic activity with a synergistic effect on downstream pathways. Balanced regulation of ROSGC1 by multiple calcium sensor proteins– and thereby, the production of cyclic GMP and downstream modulation of cyclic-nucleotide gated channels—has been well-documented in phototransduction—especially for the recovery phase in photoreceptor outer segments. In fact, S100B is critical for the recovery phase in cone photoreceptors [49] and regulates polarization activity at the photoreceptor—bipolar synapses [50]. A potential link to melatonin release as the S100B-mediated downstream effect in response to norepinephrine has been demonstrated in the pineal gland [51,52]. Notably, the central role for such opposing or synergistic regulation linked to upstream pathways such as neurotransmitter binding or activation of voltage-gated calcium channels and downstream pathways such as modulation of cyclic nucleotide gated calcium channels and altering calcium homeostasis were proposed [53].

The interaction with NCALD is also likely to mediate trafficking of S100B in response to calcium both in glia and neurons: NCALD is known to bind to cytoskeletal elements and clathrin [54] and regulate endocytosis in spinal muscular atrophy [55,56]. More recently, a role for NCALD has been proposed in neurogenesis in hippocampus [57]. It is noted that the use of BiFC allows interrogation of the NCALD-S100B interaction in a variety of cell types with different calcium transients.

Interacting partners for S100B have included receptors such as RAGE [15], TP53 and its regulatory molecules such as hdm-2 and MDM2 [58,59] and, more recently, amyloid-beta [60]. These interactions impact a variety of signaling processes associated with normal functioning and in diseased states such as cancer and Alzheimer’s disease. The calcium-dependent physiological consequences of chaperoning of S100B remain to be investigated. It is documented that HPCA can translocate brain type creatin kinase in calcium-dependent fashion [61] and such interactions play a physiological role in hippocampal neurons [62]. Thus, the calcium-dependent interaction between the versatile signaling molecules S100B and NCALD (and HPCA) potentially have significant implications for understanding the distinct roles played by S100B separated in space and time—especially in processes involving dynamic rearrangement of cell membranes, calcium-dependent relocation and secretion of S100B and delivery to targeted interacting partners. Such an understanding may be critical to evaluate S100B as a pharmacological target in the treatment of diseases.

The clinical relevance of the findings is uncharted territory, at this stage, and have to remain speculative. S100B is a versatile signaling molecule that impacts multiple physiological processes ranging from cardiomyocyte remodeling to neuronal regulation, from immune responses to cancer, as discussed earlier. It is possible that a role for this interaction may be uncovered in these processes. However, given the large overlap in expression, neurodegenerative diseases may be a good target to begin with.

## 4. Materials and Methods

### 4.1. Materials

Ovalbumin and S100B were purchased from Millipore Sigma (St. Louis, MO, USA).

### 4.2. Methods

#### 4.2.1. Protein Purification

pET 21d plasmids encoding NCALD, HPCA, NCHC, HCNC and all the mutants were transformed into ER2566 *E. coli* cells with coexpressed yeast N-myristoyl transferase. The cells were induced with Isopropyl β-d-1-thiogalactopyranoside (IPTG). Myristic acid was added to the cells to generate a myristoylated form of the proteins, which were then purified by a single-step procedure as described previously [63]. After concentration, the proteins were washed with calcium-depleted 20 mM Tris HCl (pH 7.5) to remove the bound calcium. At least three preparations of two different clones were purified and tested. 

#### 4.2.2. Membrane Overlay Assays

Two micrograms of NCALD, HPCA or a derivative were spotted onto nitrocellulose and blocked with 3% BSA in TBS-T for 1 h at room temperature. The membrane was then incubated with 80 nM of S100B in 50 mM Tris HCl, pH 7.4, 0.1% Tween-20, 150 mM NaCl, 1 mM MgSO_4_ and 1 mM DTT, in the presence of indicated concentrations of CaCl_2_ (0, 0.5 and 1 mM). After 1 h incubation, the blot was washed with the same buffer. Bound S100B was detected using its monoclonal antibody (S2532, Millipore Sigma, Burlington, MA, USA) following standard western blotting protocol and detection by chemiluminescence (Pierce ECL kit, Thermo Fisher Scientific, Waltham, MA, USA). Antibody binding was monitored by using S100B spotted onto the membrane. Intensity values were measured for each spot using Image J and corrected for background. 

#### 4.2.3. Bimolecular Fluorescence Complementation Assays

Sequences encoding NCALD and S100B were fused to sequences encoding the N-(residues 1–158) and C-terminal EYFP (residues 159–238) fragments respectively in pcDNA1 (a kind gift from Dr. Hu). Constructs were transfected into COS7 cells using calcium phosphate buffer. Standard protocols were used [48,64,65]. Briefly, the transfection mixture containing HEBS buffer (25 mM HEPES, 140 mM NaCl and 0.75 mM Na_2_HPO_4_, (pH 7.05)), 250 mM CaCl_2_ and 15 μg of plasmid DNA was incubated at room temperature for 30 min and added to COS7 cells in 35 mm dishes. After further incubation at room temperature for 30 min, fresh medium was added. Cells were grown for 24 h, and the expression of the fluorescent tagged proteins was analyzed using a C2 confocal laser scanning microscope (Nikon) equipped with Tokai-Hit stage for live imaging. Images were obtained after Hoechst addition (2 μg/mL) for nuclear staining; images were captured before and after histamine addition (45 μM). All images were captured on NIS elements using identical parameters. NIH ImageJ software [64] was used to analyze the images. Positive cell numbers were calculated and plotted in Prism. 

#### 4.2.4. Statistics

Prism (9.0) or SPSS26 was used for analyses. Statistical significance was determined by two-tailed Student’s *t* test. *, *p* < 0.05; **, *p* < 0.01; ***, *p* < 0.001. Mean ± SEM was used to represent the variations within each group.

## 5. Patents

Portions of the work submitted are currently under disclosure protection (U.S. Provisional Patent Application No. 63/084, 071).

## Data Availability

The data presented in this study are available in article and supplementary material.

## Supplementary Materials

The following are available online, Details regarding analyses of translocation described in Figure 4, including Figure S1: Estimation by freehand line tool, Figure S2: Estimation by automated area determination.

## Sample Availability

Not available.
