# Supplementary material for: Calcium-Dependent Translocation of S100B Is Facilitated by Neurocalcin Delta
Source: Molecules. 2021 Jan 5;26(1):227. doi: 10.3390/molecules26010227 (PMC7794955; doi:10.3390/molecules26010227)
Supplement: Supplementary file 1 [file molecules-26-00227-s001.pdf]

SUPPLEMENT

The images were processed in Fiji (ImageJ). Vibrations of the microscopy, mechanical relaxation and thermal fluctuation often cause sample drift in the images especially during a time course experiment. In order to compensate for this, an image stabilizer plug-in for ImageJ (http://www.cs.cmu.edu/~kangli/code/Image_Stabilizer.html) was used for each field. A correction based on the Lucas-Kanade algorithm was calculated using the DIC image. The same correction was then applied to all channels of the image stack (for instance, the DIC, the green channel for detecting the YFP signal and the blue channel to view the nucleus). The fluorescent intensity was measured. The statistical analyses for comparing the mean differences between the methods, performing regression analyses for each method, and plotting the comparison curves were performed with either Prism (9.0) or SPSS 26.

ANALYSES:

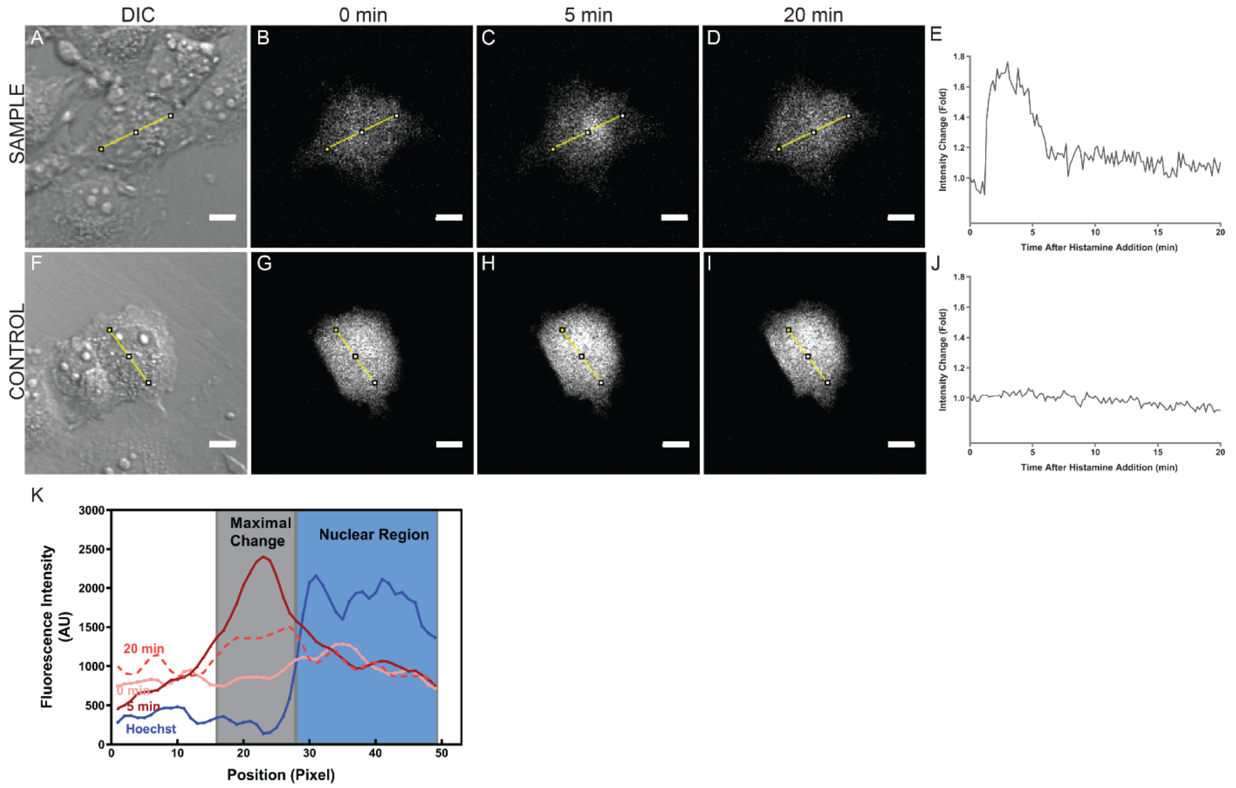

Figure S1: Estimation by freehand line tool: A line spanning the area of interest was drawn and changes in fluorescence intensity was monitored over time upon histamine addition. Images are presented for a single cell from a single experiment for HPCA-S100B complex (SAMPLE; panels A to E) or S100B-S100B complex (CONTROL; panels F to J). Panels A and F depict the respective DIC images for the Sample and Control with the ROI indicated. The TGN region was also identified based on proximity to nucleus (based on Hoechst staining) and maximal change upon histamine addition – roughly about 12-15 pixels (Panel 1K). The changes in fluorescence over this region was computed using a multi-measure tool. Scale bar = 10 μm.

To quantitate the amount of fluorescent protein at the subcellular level and its response to histamine, a tool was utilized to select the regions of interest (ROIs). Representative analyses are presented for single cells with HPCA-S100B complexes (SAMPLE) or S100B-S100B complexes (CONTROL). Similar analyses were also carried out for NCALD-S100B complexes. The granule-enriched, elevated perinuclear area corresponding to the TGN can be viewed clearly in the DIC image. The ROI was selected using the Freehand Selection tool (Figure S1; line indicated in yellow) or by automatic selection (Figure S2; area indicated in yellow) solely based on thresholding in the DIC images (A – Sample & F - Control). Then, the ROIs were saved and applied to the tested green channel. A Multi-Measure tool was utilized next to acquire the fluorescent intensity within the selected ROI across time (B-D - SAMPLE & G-I - CONTROL). The result was plotted in panels E & J.

In the freehand line method, as a first step towards quantitating the translocation, a line was drawn across each cell based on the DIC image that included the change in fluorescence in the TGN region when observable. The line would cover the nuclear region, membrane-rich perinuclear region and the rest of cytosol (indicated in yellow, Fig1A-F). Fluorescence intensity was measured for each pixel along the line at each time instance, before and after histamine addition. Measurement was carried out for both YFP and Hoechst (used to stain nuclei) along the line. In order to identify the region with maximal change in fluorescent intensity measurements relative to the nucleus, the reading of Hoechst staining was overlaid with that for YFP (Fig. 1K). The region of maximal change, where the HPCA-S100B complex concentrated in the cell, is the perinuclear area. The finding is consistent with previous studies in Hela cells [1]. The raw fluorescent intensity readings from every pixel within this region (19-27 in the representative cell) were then obtained, corrected for noise and plotted as a function of time. The values from all the pixels were then averaged and the mean is presented in figure 1E. Similar analyses were carried out with several cells transfected with the S100B-S100B complexes and the resultant curve is presented (Fig. 1J). Significant changes were observed only with transfection of both HPCA and S100B. No significant change was observed with control. Additional control, with unconjugated and intact YFP vector alone was also carried out and similar results were obtained.

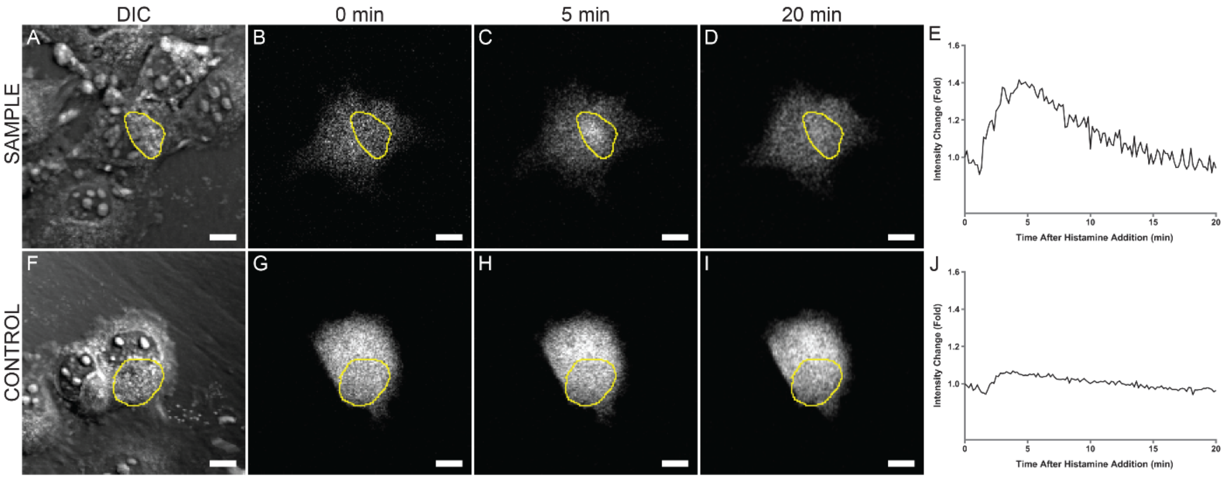

Figure S2: Estimation by automated area determination: The area of interest was identified using tools provided in ImageJ and the properties of the TGN region in the DIC image (ROI is indicated in yellow). Changes in fluorescence intensity was monitored over time upon histamine addition. Images are presented for a single cell from a single experiment for HPCA-S100B complex (SAMPLE; panels A to E) or S100B-S100B complex (CONTROL; panels F to J). The changes in fluorescence over this region was computed using a multi-measure tool. Scale bar = 10 μm.

An area tool was independently used to select the regions of interests (ROIs) and quantitate the amount of HPCA at the subcellular level and its response to histamine. The representative results from a single cell are shown in Fig. S2. The granule-enriched elevated perinuclear area can be viewed clearly in the DIC image and were selected as the ROI based on tools provided in ImageJ for thresholding (marked by a yellow boundary) in cells with both HPCA-S100B complexes (SAMPLE) and S100B-S100B complexes (CONTROL). The ROIs were saved and applied to the images to measure the fluorescent intensity within the selected ROI across time (Panels B through D, G through I) using a Multi-Measure tool. The result obtained from the representative cells was plotted (Panels F, J). Upon histamine addition, a sharp increase in the fluorescent intensity followed by a gradual decrease within the tested ROIs was detected in the cells with HPCA-S100B complexes, which indicates a translocation of the complex into the ROI. However, in the control cell, the fluorescent intensity from the ROIs stayed comparably similar during the observed time frame (50 seconds before adding histamine to 20 minutes after the addition).

Data obtained by the two different methods were evaluated statistically. The mean fluorescent intensity measurements for each sample were recorded for 121 times ranging from 0 to 20 min. The mean values across each of the 121 time-intervals for the cells were next calculated independently for each sample. Seven samples were analyzed by both methods. The mean total intensity for all of the 121 line method estimates was 1.45 (SD = 0.23), and the mean total intensity for all of the 121 area method estimates was 1.25 (SD = 0.12), Welch's $t'(178) = 8.74$, Cohen's $d = 1.09$, $P < .001$. The mean intensity measurement estimated by the line method was thus significantly higher than the mean intensity measurement estimated by the area method. This observation is consistent with the fact that the line is drawn subjectively based on observable intensity differences. A product-moment correlation was calculated between the 121 mean intensity values for both methods to determine the magnitude of the similarity between the curve shapes. The significant correlation was 0.95, $P < .001$, indicating high similarity and suggesting that both line and area methods might be reflecting similar curves over time. It is noted that some differences in the kinetics are observed between the two methods and will be investigated. Data thus collected from several cells over multiple transfections were pooled for analyses by the line method and presented in figure 4.

The authors are also grateful to Professor Robert Steer (Department of Psychiatry, Rowan School of Osteopathic Medicine) for help with the statistical analyses.
